# Kombucha Tea: A Functional Beverage and All its Aspects

**DOI:** 10.1007/s13668-025-00658-9

**Published:** 2025-05-24

**Authors:** Begum Onsun, Kadriye Toprak, Nevin Sanlier

**Affiliations:** Department of Nutrition and Dietetics, Faculty of Health Sciences, Ankara Medipol University, Ankara, Turkey

**Keywords:** Kombucha tea, Functional foods, Scoby, Fermented beverages

## Abstract

**Purpose of review:**

The increasing interest in functional foods and beverages worldwide is driven by rising living standards, advancing technology, and heightened health awareness. Kombucha tea, a fermented beverage produced from sweetened tea and a symbiotic culture of bacteria and yeast (SCOBY), is a prominent example within this category. This review explores the definition, bioactive components, and health benefits of kombucha, emphasizing its potential roles as a functional beverage in the prevention and management of various diseases.

**Recent findings:**

The fermentation process of kombucha tea, typically lasting up to 14 days, results in the transformation of sugar into ethanol and acetic acid, contributing to its distinctive tangy flavor. Kombucha contains bioactive compounds such as organic acids, antioxidants, and probiotics, which are linked to potential health benefits including improved digestive health, enhanced immune function, and antioxidant activity. Recent advancements in sustainable production methods and innovative formulations have further contributed to the increasing popularity of this beverage.

**Summary:**

Kombucha tea, originating in Northeast China with a history of over 2,000 years, is increasingly recognized for its potential health-promoting effects. Its production through traditional fermentation methods combined with modern innovations underscores its value as a functional beverage with the potential to support health and well-being. This review assesses the roles of kombucha in maintaining human health, considering its use as a complementary strategy for the prevention and management of diseases due to the bioactive components it contains.

**Graphical Abstract:**

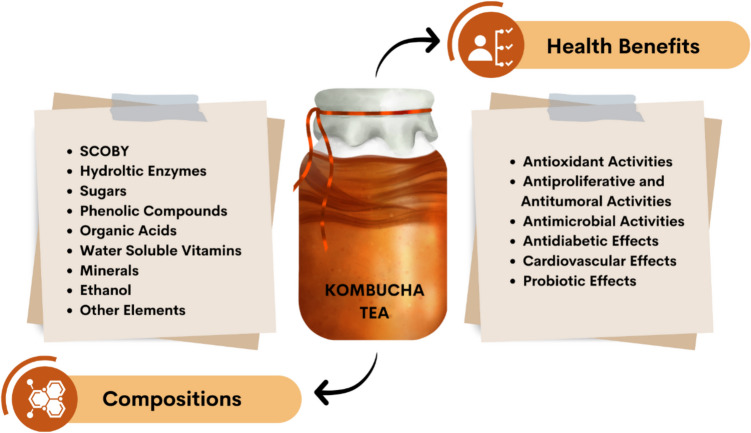

## Introduction

In contemporary society, the aging of the population, increasing urbanization, shifts in income levels, and advancements in technology have collectively exerted significant influence on individuals’ efforts to improve their living standards. Expanded access to health services, the evolution of dietary habits, and the growing emphasis on physical activity are fostering aspirations within society to attain healthier and more fulfilling lives [1, 2]. This surge of “health consciousness” has inspired various changes in individuals’ dietary habits. At the same time, the clear importance of adopting an adequate and balanced nutrition plan to optimize both physical and mental health is steering consumers towards more nutrient-rich food choices [1–3]. This trend is steadily increasing the demand for foods that offer additional health benefits. The concept of “functional foods” has gained popularity as a result of such foods not only being consumed for satiety, energy, and the meeting of nutritional needs but also for their positive contributions to health by aiding in the reduction of the risk of developing various diseases [4, 14]. However, there is no universally accepted definition of functional foods. The European Union’s Functional Food Commission, in affiliation with the European Food Information Council (EUFIC), has defined functional foods as those that contain bioactive components beyond the needs of basic nutrition, offering health benefits to enhance both physical and mental well-being while also having the potential to reduce the risk of certain health problems [5, 14, 15]. Different countries may utilize the term “functional foods” in various ways within the scope of relevant legislation [4, 5, 14, 15]. Functional foods also include beverages, which play a significant role in this category [6]. Notably, fermented beverages warrant particular attention due to their unique health-promoting benefits, which extend far beyond merely providing hydration. These beverages contain bioactive components that have been demonstrated to have positive effects on health [6, 7]. As a fermented beverage, kombucha tea is of significant value within the larger category of functional foods, particularly due to its probiotic characteristics and the beneficial effects it exerts on intestinal health [8, 9]. Considering the general rates of production and consumption, the United States constitutes the most significant and dynamic market for functional foods, with a market share exceeding 50% [10]. The overall budget devoted to functional foods is predicted to reach approximately 280 billion US dollars in 2025, with an annual growth rate of about 8% [11]. At the same time, there has been a noticeable increase in the quantity and diversity of functional foods available in Türkiye since 2005 [12]. In this context, kombucha has gained significant popularity in recent years in both Western countries and emerging markets such as Türkiye. This trend, driven by kombucha’s probiotic properties and perceived benefits for intestinal health, has coincided with the broader growth of the functional food market as a whole [13–15]. In this review study, the fermentation process, composition, and possible health effects of kombucha tea are discussed. A comprehensive evaluation is presented in light of the findings currently available in the scientific literature. The effects of the bioactive components contained in kombucha on health and its position among other functional beverages are examined and current research in this field is discussed.

## Kombucha Tea: A Complex Fermented Beverage

Kombucha, a beverage with origins in East Asia that has been consumed for centuries due to its perceived health benefits, has long held prominence in traditional medicine for its potential in promoting healthy digestive and immune system functions [16–19]. Recent scientific research has confirmed that kombucha may positively impact intestinal health, metabolic balance, and overall well-being, benefits attributable to its probiotics, organic acids, and antioxidants [20–23]. Findings such as these have contributed to the rapid rise in popularity of kombucha as a functional food, allowing it to transcend its traditional identity as merely a fermented beverage [17, 20]. This section of the study summarizes the history and production stages of kombucha tea according to the currently available literature.

### History of Kombucha Tea

Tea is known to have been used as one of the oldest remedies in China 5,000 years ago for its stimulating and detoxifying effects, aiding in the elimination of the harmful effects of alcohol and toxins in the body, alleviating joint pain, improving blood and urine flow, and enhancing immunity against diseases [17, 18, 22]. Kombucha, believed to possess special healing properties for a healthy and long life, is a fermented tea beverage originating from Far Eastern Asia that has also been consumed for thousands of years [18, 19, 23]. In Japanese, the word “kombucha” is formed by the combination of “kombu,” referring to the broad-leaved seaweed *Laminaria japonica*, and “cha,” meaning tea [24]. The use of kombucha began in China around 220 BCE, and it was later introduced to Japan for the treatment of an emperor. Subsequently, with the expansion of trade routes, its recognition started to grow in Russia and other European countries [25]. Due to its suggested therapeutic effects, its role in improving health, and alleged abilities such as delaying aging, kombucha has become a popular functional food in recent years, particularly in the Western hemisphere and especially in the United States [26].

### Production of Kombucha Tea

**Fig. 1:**
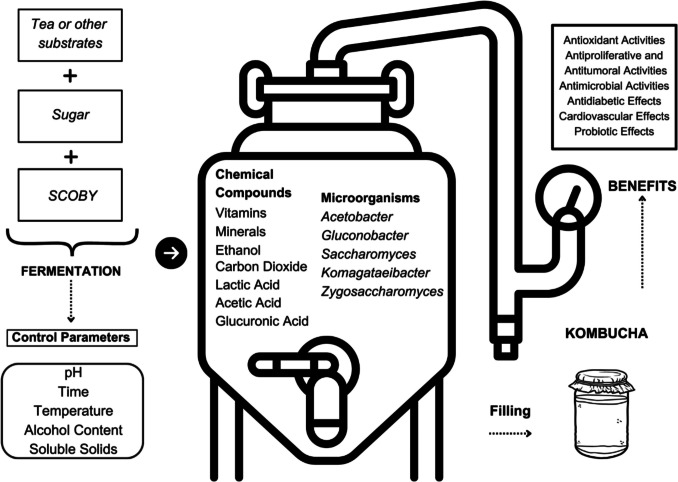
Production stages and control parameters for Kombucha [34]

For the traditional production of kombucha, an initial base tea is chosen, typically using dried black tea leaves of *Camellia sinensis*. Subsequently, sugar is added to the selected tea to serve as a substrate, facilitating the fermentation of the tea by bacteria and yeast [27]. The yeast necessary for this fermentation process is referred to as SCOBY, an acronym for “symbiotic colony of bacteria and yeast” [27, 28]. The collaborative activities of the bacteria and yeast in the preparation of kombucha are defined as symbiosis. While yeast species in the colony metabolize sucrose into fructose, glucose, ethanol, and acids, bacterial species consume ethanol and simple sugars as fuel, producing organic acids such as gluconic acid, glucuronic acid, and acetic acid. Throughout the fermentation process, the bacteria and yeast continue to work together, ensuring the progression of the fermentation [23, 27, 28, 55]. Acetic acid bacteria such as species of the genera *Acetobacter*, *Gluconobacter*, and *Komagataeibacter* are commonly used in these symbiotic cultures together with various osmophilic yeast species such as those belonging to *Saccharomyces* or *Zygosaccharomyces* [29]. Due to the potential for high levels of acetic acid production during the fermentation process, regular monitoring of the pH level is crucial, and it is recommended to halt fermentation when the pH reaches 4.2 [30]. To prevent the inactivation of the microorganisms, the prepared tea should be cooled to room temperature [31]. A study conducted by Carvalhes et al. demonstrated that storing kombucha in a refrigerator will reduce the viability of beneficial microorganisms within the tea while potentially promoting the proliferation of undesirable microorganisms [32]. Additionally, pasteurization is a crucial process for ensuring food safety and preventing problems that might arise during storage. Through pasteurization, excessive alcohol production and acidification of the beverage are avoided [33]. For this reason, it is recommended that the final obtained beverage be filtered after a pasteurization process and stored in closed containers, allowing for the removal of cellulose residues and unwanted masses of microorganisms in the suspension [34]. The production stages and control parameters for kombucha are summarized in Fig. 1.

## What Bioactive Substances are Present in Kombucha Tea?

The primary substrates preferred for producing kombucha are black, green, white, or oolong tea and white sugar. The fermentation of these substrates results in the formation of micronutrients such as polyphenols, organic acids, active enzymes, water-soluble vitamins, and various other chemical compounds [35]. The quantity, contents, and antioxidant activities of the bioactive compounds in kombucha vary according to several different factors. The type of tea plant chosen for the substrate, the duration of fermentation, the types of microorganisms involved in the SCOBY, the metabolic activities of those microorganisms, and the type and amount of sugar used have all been found to influence the quantity of bioactive substances in the final product [36].

### Polyphenols

Extensive research has confirmed that tea, one of the primary substrates used in the preparation of kombucha, is rich in polyphenols that contribute to human health [37, 56]. Moreover, during the kombucha fermentation process, the symbiotic relationship between the yeast and bacteria in the SCOBY leads to the production of various bioactive compounds in the substrate and an increase in the phenolic contents of the raw material [37]. This increase occurs as a result of the microorganisms releasing bound polyphenols from the tea leaves during fermentation, producing new phenolic compounds as a result of microbial metabolism [38]. Specifically, β-glucosidase, an enzyme produced by yeasts and bacteria, hydrolyzes glycosidic bonds, thereby releasing free polyphenols from their bound forms [39]. Additionally, tannase enzymes break down tannins into simpler phenolic compounds, enhancing their bioavailability [40]. The metabolic activity of acetic acid bacteria and yeasts further contributes to the breakdown of complex polyphenolic structures, resulting in a higher concentration of bioactive and antioxidant compounds [41, 42]. Furthermore, fermentation-induced acidification facilitates polyphenol extraction from tea leaves, leading to a more enriched bioactive profile of Kombucha tea [43, 44]. Several studies have demonstrated that the fermentation process significantly enhances the polyphenol content and antioxidant capacity of Kombucha, further supporting its potential health benefits [43, 44]. In the study conducted by Ivanišová et al. [43], it was observed that the total polyphenol contents of fermented kombucha were higher compared to non-fermented black tea. For kombucha fermented with black tea as the substrate, it has been reported that the release of catechins from acid-sensitive cells during fermentation contributes to the increased polyphenol contents of the resulting tea. Furthermore, Zhou et al. [44] reported that the fermentation of kombucha in the presence of tea residues significantly increased the total antioxidant activity and polyphenolic contents of the final beverage. During the production stage, the residue generated when using green tea as a substrate exhibited more significant polyphenolic effects than black tea. Therefore, it was concluded that the types and quantities of polyphenols in kombucha may vary depending on the variety of tea used [44].

### Organic Acids

Organic acids are also among the valuable bioactive components formed during the production of kombucha tea. The production of organic acids during the fermentation process lowers the pH of the substrate environment [45]. This decrease in pH contributes to the development of kombucha’s sour taste. Critical organic acids involved in the formation of this sour taste include acetic acid, gluconic acid, and citric acid [46]. In a study conducted by Kaewkod et al. [47], black tea was used as a substrate for kombucha fermentation. After a 5-day fermentation period, the concentrations of various organic acids were measured as follows: acetic acid at 11.15 g/L, glucuronic acid at 1.58 g/L, gluconic acid at 70.11 g/L, ascorbic acid at 0.70 g/L, and D-saccharic acid-1,4-lactone (a derivative of glucuronic acid) at 5.23 g/L. These findings indicated that the fermentation process significantly increased the content of these beneficial organic acids in the kombucha. Furthermore, the organic acids produced in kombucha, including those listed here, were found to be effective against pathogenic enteric bacteria such as *Escherichia coli* and specifically *E. coli* O157:H7, *Salmonella* Typhi, *Shigella dysenteriae*, and *Vibrio cholerae*. In another study by Neffe-Skocińska et al. [48], analysis of the glucuronic acid contents of kombucha showed an increase at all temperatures during processing. However, the highest fermentation level was observed at 25 °C on the 10 th day. This literature review has revealed the lack of adequate studies for a full understanding of the types and quantities of organic acids formed in kombucha production, emphasizing the need for further research.

### Water-Soluble Vitamins

Another crucial characteristic of kombucha that contributes to improved human health is the presence of trace amounts of water-soluble vitamins [49]. Kombucha has been reported to contain water-soluble vitamins, including vitamin C, which is essential for collagen synthesis in connective tissue, functions as a powerful antioxidant by neutralizing free radicals, and enhances immune system functionality to protect against infections [49–51]. And it has also been reported to contain water-soluble B-group vitamins, including B_1_, B_6_, and B_12_, which play a pivotal role in energy metabolism, support nervous system functions, act as co-factors in neurotransmitter synthesis and cell metabolism, and are essential for red blood cell production [49, 52, 53]. The vitamin content in kombucha, particularly vitamin C and B-group vitamins, varies depending on the fermentation process and ingredients used. Mousavi et al. [54] was determined the vitamin C content in kombucha tea 25 mg/L after 10 days of fermentation. One study reported that the vitamin C content in kombucha can reach up to 151 mg/L [55]. Similarly, in a study B-group vitamins (B_1_, B_6_, and B_12_) were detected in concentrations ranging from 0.1–0.5 mg/mL [54] while the B-group vitamins were detected in the following concentrations: vitamin B_1_ at 0.74 mg/mL, vitamin B_6_ at 0.52 mg/mL, and vitamin B_12_ at 0.84 mg/mL in another study [55]. In a study by Frolova et al. [56], formulations of kombucha tea enriched with vitamins and inulin, including strawberry and lemon variations, were developed. The resulting beverages were stored in dark glass bottles to prevent the degradation of vitamins and preserve their contents during long-term storage. The authors concluded that adding B vitamins and inulin to kombucha resulted in an acceptable organoleptic profile, suggesting the possibility of creating new customized beverages tailored to individual needs. However, the research to date on the vitamin and mineral contents of kombucha remains limited by the paucity of studies, with findings varying depending on factors such as the type of tea used and the fermentation time [57]. In particular, significant differences have been reported between the micronutrient contents of kombuchas made from green and black tea [58]. Further research is needed to optimize the nutritional value of kombucha. Comparative analyses with other fermented beverages are also required.

### Ethanol

Another significant compound formed during the production of kombucha is ethanol. The ethanol generated in kombucha production has sparked debates regarding the classification of this beverage as non-alcoholic. Kombucha is categorized as a “non-alcoholic beverage” as long as its alcohol content does not exceed a specific threshold value [59]. According to the literature, the ethanol contents of the majority of produced kombuchas are above 0.5% (w/w), and according to regulations in many countries, beverages with such values should be categorized as “alcoholic beverages” [60]. Considering that variations in the production process can lead to changes in the final product’s contents and quantities of different compounds, determining the end product’s ethanol content will assist in making more accurate decisions regarding the classification of the beverage as alcoholic or non-alcoholic.

## Effects of Kombucha Tea on Health

The success of kombucha in meeting consumer expectations with both its high sensory value and its various health benefits has been a major factor in its use for over 2,000 years [61]. Studies showing that kombucha has various antioxidant, antimicrobial, antiproliferative, antitumoral, and antidiabetic properties and other beneficial effects on health are listed in Table 1. In this section, the various health effects of kombucha are summarized based on a review of the literature.

**Table 1:** Studies on the relationship between Kombucha tea and its health effects

Health Effect	Year	Study Design	Results
Antioxidant Activities [43]	2019	in vitro	It has been observed that the total polyphenol content of fermented Kombucha tea is higher than that of unfermented black tea. Kombucha tea fermented using black tea, the release of catechins from acid-sensitive cells as a result of fermentation increases the polyphenol content of the resulting tea
Antioxidant Activities [44]	2022	in vitro	It has been determined that when green tea is used as a substrate during the production stage, the residue formed provides a stronger antioxidant and polyphenolic effect compared to black tea
Antioxidant Activities [70]	2023	in vivo & in vitro	Strawberry and lemon formulations of Kombucha tea enriched with vitamins and inulin have been developed. It has been stated that the added crushed strawberry and lemon leaves can increase the antioxidant level of the final product
Antimicrobial Activities [47]	2019	in vitro	In this study, where black tea was used as a substrate, it has been shown that the content of acetic acid, glucuronic acid, gluconic acid, ascorbic acid, and succinic acids reached high levels after 5 days of fermentation. These organic acids formed in Kombucha have been found to be effective against pathogenic enteric bacteria such as Escherichia coli, E. coli O157:H7, Salmonella Typhi, Shigelladysenteriae and Vibrio cholera
Antimicrobial Activities [48]	2017	in vivo & in vitro	The analysis of Kombucha beverages revealed an increase in the content of the organic acid"gluconic acid,"contributing to antimicrobial properties during the fermentation process
Antimicrobial Activities [73]	2018	in vivo & in vitro	In this study, the antimicrobial activity of Kombucha Tea against microorganisms such as E. Coli, Bacillus subtilis, Staphylococcus aureus, Klebsiella pneumoniae, Proteus vulgaris and Candida albicans was examined, and the antimicrobial activity against all microorganisms tested after 7 days of fermentation was associated with the acetic acid content of Kombucha.
Antimicrobial Activities [77]	2023	in vivo & in vitro	It has been stated that Kombucha tea cultures prepared with black tea can be used as an antifungal agent in the treatment of vulvovaginal candidiasis
Antitumoral Activities [81]	2021	in vitro	It has been determined that Kombucha has anticancer activity against colorectal cancer by increasing the activity of the antitumor agent doxorubicin in the treatment of colorectal cancer
Antitumoral Activities [83]	2013	in vitro	It has been stated that Kombucha tea reduces the development and spread of prostate cancer cells by suppressing the expression of angiogenesis stimulants. Therefore, it suggests that it may be used as a useful agent in inhibiting prostate adenocarcinoma
Antitumoral Activities [73]	2018	in vivo & in vitro	It was observed that Kombucha tea prepared with yarrow showed antiproliferative properties against rhabdo myosarcoma and cervical carcinoma cells in humans
Antitumoral Activities [47]	2019	in vitro	Kombucha prepared from green tea and black tea has been reported to have antioxidant activity and selective toxicity on Caco-2 colorectal cancer cells
Anti-diabetic Effects [88]	2022	in vivo	In a study conducted on mice, Kombucha tea was shown to have positive effects in the prevention and treatment of diabetes. It has been stated that Kombucha balances the intestinal microbiota by helping to reduce harmful bacteria and increase beneficial bacteria, which may reduce blood glucose levels
Anti-diabetic Effects [86]	2023	in vitro	It has been determined that the extracts used exhibit inhibitory activities against enzymes responsible for the onset and progression of diabetes and may have antidiabetic effects
Anti-diabetic Effects [89]	2023	in vivo	In a pilot study conducted on humans, Kombucha tea was associated with positive effects on reducing blood sugar levels in individuals with Type 2 Diabetes
Cardiovascular Effects [91]	2020	in vivo	It supports that kombucha tea consumption may be effective in preventing problems caused by high cholesterol diet consumption
Cardiovascular Effects [92]	2020	in vivo	Kombucha tea is a functional food containing high levels of antioxidants that can delay the formation of atherosclerotic lesions. The antioxidants it contains have been shown to prevent the progression of atherosclerotic lesions
Cardiovascular Effects [93]	2022	in vivo	It was stated in the case report that cardiotoxic effects could be seen

### Antioxidant Effects of Kombucha Tea

Kombucha has various antioxidant properties, with the primary bioactive components responsible for its antioxidant effects being polyphenols and glucuronic acid [62]. Polyphenols are a class of antioxidant compounds that have the ability to neutralize free radicals, thereby reducing cellular damage [63]. In addition, they have been shown to reduce the risk of chronic disease by suppressing inflammation [64]. Glucuronic acid, another polyphenol of interest, plays a role in the detoxification of the body, contributing to the reduction of oxidative stress [65]. The types and transformations of polyphenolic compounds vary depending on the SCOBY content of the kombucha, the duration of fermentation, and the tea substrate used. The majority of its high polyphenol contents consist of flavonoids. Flavonoids can protect cell membranes from oxidative stress by inhibiting lipid peroxidation [66]. Due to the potent antioxidant properties of polyphenols, the consumption of kombucha is particularly recommended for individuals exposed to oxidative stress [67]. In a study by Kaewkod et al. [47], kombucha prepared using green, black, and oolong teas inhibited pathogenic enteric bacteria and exhibited antioxidant activity and selective toxicity against colorectal cancer cells. These findings showed that kombucha can both neutralize free radicals and support cellular defense mechanisms due to its polyphenols and organic acids [47]. However, excessively long fermentation durations may lead to the accumulation of organic acids, which could negatively impact the taste profile and the bioavailability of beneficial compounds [68]. One study found that the antioxidant activity of kombucha changed according to the fermentation period and reached the highest level on the 7th day (93.8%). A slight decrease was subsequently observed as the duration increased, falling to 93.6% on the 11th day. Furthermore, as the fermentation progressed, the pH decreased from 5.93 to 3.65 and the color changed from dark brown to lighter shades [69]. Numerous studies have demonstrated the antioxidant potential of kombucha [38, 43, 44, 70, 71]. However, differences in final products due to variations in fermentation conditions, tea substrates, and analytical methods highlight the necessity of further standardized research to confirm the bioactive properties of kombucha and optimize its health benefits [72].

### Antimicrobial Effects of Kombucha Tea

During the kombucha fermentation process, various bioactive compounds with antimicrobial potential are produced [73]. It is particularly thought that the primary functional activities of the generated organic acids are responsible for microbiological safety. Therefore, pH is an essential parameter in the fermentation process [74]. In acidic foods with pH values between 4.0 and 4.5 and in highly acidic foods with pH of < 4.0, the development of pathogenic microorganisms is not observed. Foods within this pH range are considered microbiologically safe [75]. The Food Code of the US Food and Drug Administration (FDA) recommends a critical threshold value for fermentation at a pH of ≤ 4.2. Current regulations state that the pH value should be between 2.5 and 4.2 for kombucha tea to be considered microbiologically safe. It is advised to discard cultures that do not reach such values within 7 days due to the risk of contamination [76]. In a study conducted by Vitas et al. [43], the antimicrobial activity of kombucha against microorganisms such as *E. coli*, *Bacillus subtilis*, *Staphylococcus aureus*, *Klebsiella pneumoniae*, *Proteus vulgaris*, and *Candida albicans* was investigated. The antimicrobial activity observed against all tested microorganisms after a 7-day fermentation period correlated with the acetic acid contents of the kombucha. A study published in 2023 further suggested that kombucha cultures prepared with black tea could be used as antifungal agents in the treatment of vulvovaginal candidiasis [77]. Another study observed potential toxic effects and the possibility of drug resistance with the current treatment methods for fungal diseases. The need for new and safer alternative treatments was accordingly emphasized [78]. Kombucha is considered a candidate for an alternative antimicrobial source and is also thought to have promise as an alternative treatment agent in combating the increasing threat of antibiotic resistance [79]. Another study investigating kombucha’s antimicrobial activities found that fermentation conditions and tea types impacted the microbiological properties and effects of the final product [80]. This indicates that the antimicrobial effects of kombucha are not consistent across different production conditions and inconsistencies are present in the literature. More controlled studies are needed in this field to better understand the effect of different variables in kombucha production and obtain more reliable results.

### Antiproliferative and Antitumoral Effects of Kombucha Tea

The combination of traditional functional products with chemotherapy in cancer treatment constitutes a promising research area. According to a recent study, kombucha exhibited anticancer activity against colorectal cancer by enhancing the activity of the antitumor agent doxorubicin [81]. Recent scientific evidence suggests that kombucha may help rebalance the pH levels that tend to increase during the course of the disease in cancer patients. Additionally, lactic acid produced through fermentation may alleviate L-lactic acid deficiency in the connective tissues of cancer patients [82]. Another study noted the antiproliferative effects of kombucha prepared with yarrow against rhabdomyosarcoma and cervical carcinoma cells in humans [73]. Furthermore, kombucha was shown to suppress the expression of angiogenesis stimulators, reducing the development and spread of prostate cancer cells. Therefore, it could be used as a beneficial agent in inhibiting prostate adenocarcinoma [83]. A recent study showed that kombucha exerted potent cytotoxic and apoptosis-inducing effects against HepG2 liver cancer cells with no significant growth inhibition in normal cell lines [84]. In addition, its IC_50_ values were found to differ between cell models, indicating potential variability in its efficacy against different cancer types. These findings suggest that while kombucha may have anticancer properties, further research is needed to clarify its effects in different cancer models and determine optimal dosing strategies [84]. Furthermore, its anticancer effects have primarily been evaluated with in vitro studies to date; therefore, it is not certain that the same effects could be achieved in vivo. More controlled clinical studies are needed to clarify the biological activities, mechanisms of action, and safe doses of kombucha in cancer treatment [81–85].

### Antidiabetic Effects of Kombucha Tea

Researchers have suggested kombucha as an alternative option in efforts to design new strategies for the improvement of blood glucose control. In an in vitro study, inhibition of both α-amylase and α-glucosidase was observed with the extracts used. It was determined that these kombucha extracts displayed inhibitory activities against the enzymes responsible for the onset and progression of diabetes and could have antidiabetic effects [86]. A study involving a rodent model of hyperglycemia concluded that kombucha improved the adverse effects of diabetes, showing significant beneficial health effects [87]. Kombucha tea is suggested to be effective in both preventing and treating diabetes [26, 87]. A study of mice showed the positive effects of kombucha in preventing and treating diabetes, and the researchers associated those positive effects with the positive effects of kombucha on the gut microbiota. Kombucha was shown to balance the gut microbiota by reducing harmful bacteria and increasing beneficial bacteria, which could lead to a decrease in blood glucose levels [88]. In a pilot study conducted with humans in 2023, kombucha was found to have potential positive effects via the reduction of blood sugar levels in individuals with type 2 diabetes [89]. Although most recent studies support the antidiabetic effects of kombucha, the optimal dose and long-term effects are unclear because the contents of kombucha and the obtained results vary depending on the fermentation time and microbial composition. Therefore, more comprehensive clinical trials with standardized protocols are needed.

### Cardiovascular Effects of Kombucha Tea

Cardiovascular diseases are among the leading causes of death both in Türkiye and worldwide [90]. While pharmaceutical treatments are commonly used in these cases, functional foods might also be effective [85, 91]. Kombucha is a functional food containing high levels of antioxidants that can delay the formation of atherosclerotic lesions. Its antioxidants were shown to prevent the progression of atherosclerotic lesions [92]. Doudi et al. [91], in a study conducted with rabbits, concluded that consuming kombucha may effectively prevent problems arising from a high-cholesterol diet. In addition to these beneficial effects, a case report published in 2022 stated that cardiotoxic effects could also be observed. A female patient lacking other chronic medical conditions presented to the emergency department with complaints of nausea and vomiting following the ingestion of homemade kombucha tea. Despite the diagnosis of ST-segment elevation myocardial infarction (STEMI) in the cardiology department, the patient died and lactic acidosis was detected in blood sample analyses [93]. This case underscores the importance of meticulous preparation in the production stages of kombucha and also indicates the need for more detailed investigations into its cardioprotective properties.

### Probiotic Effects of Kombucha Tea

Kombucha is categorized as a symbiotic beverage due to its positive effects on the gastrointestinal microbiota, attributed to its short-chain fatty acids and metabolites [94]. The ingestion of kombucha was shown to enhance digestive health and elevate overall vitality by encouraging the proliferation of beneficial microorganisms within the intestinal tract. The probiotic bacteria present in kombucha promote the growth of *Lactobacillus* and *Bifidobacterium* species, particularly in the intestinal tract [95]. Thus, kombucha has become a popular choice for those seeking to enhance their digestive health [96]. However, despite recent findings indicating potential benefits of kombucha for intestinal health, the literature on this subject is somewhat contradictory [93, 95, 97]. While Lobo et al. [97] described kombucha as a significant source of probiotics, Vargas et al. [95] did not categorize kombucha as a true probiotic beverage due to the incomplete understanding of the effects of produced metabolites on the human microbiota and the inability to standardize the contents of the final products due to external factors. Another study indicated no changes in intestinal flora after administration of kombucha in both in vivo and in vitro experiments [93]. Further research is needed to enhance our comprehension of the probiotic benefits of kombucha, particularly with regard to the impact of different fermentation processes and ingredients on the microbiota of the final product. Such studies will facilitate more comprehensive assessments and evaluations of the probiotic attributes of kombucha.

## Conclusion

Kombucha tea is a traditional functional beverage with a constantly expanding range of applications. The recent increase in its popularity has prompted comprehensive investigations of the unique aspects of this functional beverage. In this review, kombucha’s history, production processes, bioactive components, microbial diversity, and relationships with health have been analyzed as a result of an extensive literature review to better understand the functionality of this traditional beverage. Various studies have suggested that, in light of the positive effects of its bioactive components on human health, kombucha could be utilized as a supportive measure in the prevention and treatment of numerous diseases. However, despite these promising findings, there are inconsistencies in the literature regarding its bioactive properties, optimal fermentation conditions, and health effects. Variations in study methodologies, tea substrates, and microbial compositions contribute to these differences in results. While some studies have highlighted its antioxidant, antimicrobial, and probiotic effects, others suggest that prolonged fermentation or specific compositions may reduce its efficacy. Therefore, further standardized and controlled studies are needed to clarify these conflicting findings and establish clear guidelines for the consumption of kombucha with the aim of obtaining optimal health effects.

### Limitations

Despite the promising evidence regarding the health benefits of kombucha tea, it is important to note that the existing research in this area is limited and conflicting. Primarily, as most studies are based on in vitro or animal models, it is unclear whether these results can be generalized to humans. Furthermore, the lack of standardization in the preparation of kombucha, including variations in the type of tea used, fermentation time, microbial content, and production conditions, complicates the determination of the health effects of the bioactive constituents contained in kombucha. While some studies report high antioxidant and probiotic activities [38, 43, 44, 70, 71, 97], other studies demonstrate that these effects decrease or disappear depending on fermentation time or compositional differences [69, 72, 93, 95]. In addition, significant inconsistencies have been observed between studies investigating vitamin and organic acid levels [48, 57, 58]. Furthermore, it has been reported that findings regarding the antimicrobial and antiproliferative effects of Kombucha cannot be replicated under some production conditions [80–85]. Consequently, randomized controlled human studies using more standardized products and protocols are essential to obtain accurate and generalizable results regarding the health effects of Kombucha.

### Future Perspectives

Considering the increasing awareness of the importance of a healthy lifestyle and the accompanying increased inclusion of functional foods in individual diets, it is anticipated that kombucha will be increasingly more preferred as a functional beverage in the future. Beverage industries may facilitate the production of various formulations of kombucha with the aim of assisting in the treatment of many health problems. If innovative formulations and profiles are combined with sustainable production practices, the application areas of kombucha may expand, increasing its popularity as a functional beverage.

## Key References

- Bassyouni RH, Ahmed FA, Ismaiel AA, et al. In-vitro antifungal activities of kombucha tea culture supernatant combined with voriconazole against vulvovaginal candidiasis clinical isolates. J Health Sci Med Res. 2023;41(4):2023933.
  - ⚬ This article highlights the potential antifungal properties of kombucha tea cultures when used in combination with voriconazole against resistant *Candida* strains isolated from cases of vulvovaginal candidiasis. The findings show that kombucha significantly reduces the minimum inhibitory concentration of voriconazole, enhancing its efficacy even against biofilm-forming strains. These results underscore kombucha’s potential as a complementary functional beverage in antifungal therapy, providing a novel perspective on its health benefits and expanding its applications beyond its traditional use.
- Xu S, Wang Y, Wang J, Geng W. Kombucha reduces hyperglycemia in type 2 diabetes of mice by regulating gut microbiota and its metabolites. Foods. 2022;11(5):754.
  - ⚬ This study provides a comprehensive investigation of the hypoglycemic mechanisms of kombucha, focusing on its modulation of gut microbiota in a mouse model of type 2 diabetes mellitus. The kombucha intervention enhanced the abundance of SCFA-producing bacteria, reduced the counts of pathogenic bacteria, and improved intestinal barrier integrity. These changes attenuated inflammation and insulin resistance while promoting islet β-cell function through gastrointestinal hormone secretion. This study offers valuable insight into the antidiabetic potential of kombucha, reinforcing its status as a functional beverage for the prevention and management of diabetes.
- Frolova Y, Vorobyeva V, Vorobyeva I, Sarkisyan V, Malinkin A, Isakov V, Kochetkova A. Development of fermented kombucha tea beverage enriched with inulin and B vitamins. Fermentation. 2023;9(6):552.
  - ⚬ This article explores the development of kombucha beverages enriched with functional ingredients such as inulin and B vitamins, providing insights into the formulation and production processes. By examining the fermentation dynamics, including changes in pH, organic acids, and antioxidant activity, it highlights the potential of kombucha as a nutritionally enhanced product. The beverage’s ability to meet a significant portion of the recommended daily intake for B vitamins and inulin reflects its value as an enriched functional drink. This study was instrumental in advancing the understanding of kombucha’s versatility and appeal as a health-promoting beverage.

## Data Availability

No datasets were generated or analysed during the current study.
